# It Is Always on Your Mind: Experiences and Perceptions of Falling of Older People and Their Carers and the Potential of a Mobile Falls Detection Device

**DOI:** 10.1155/2013/295073

**Published:** 2013-12-23

**Authors:** Veronika Williams, Christina R. Victor, Rachel McCrindle

**Affiliations:** ^1^Primary Care Clinical Trials Unit, Department of Primary Care Health Sciences, 23-38 Hythe Bridge Street, Oxford OX1 2ET, UK; ^2^Gerontology and Public Health, School of Health Sciences and Social Care, Brunel University, Uxbridge, Middlesex UB8 3PH, UK; ^3^Computer and Human Interaction, School of Systems Engineering, University of Reading, Reading RG6 6AY, UK

## Abstract

*Background*. Falls and fear of falling present a major risk to older people as both can affect their quality of life and independence. Mobile assistive technologies (AT) fall detection devices may maximise the potential for older people to live independently for as long as possible within their own homes by facilitating early detection of falls. *Aims*. To explore the experiences and perceptions of older people and their carers as to the potential of a mobile falls detection AT device. *Methods*. Nine focus groups with 47 participants including both older people with a range of health conditions and their carers. Interviews were audio recorded, transcribed verbatim, and thematically analysed. *Results*. Four key themes were identified relating to participants' experiences and perceptions of falling and the potential impact of a mobile falls detector: cause of falling, falling as everyday vulnerability, the environmental context of falling, and regaining confidence and independence by having a mobile falls detector. *Conclusion*. The perceived benefits of a mobile falls detector may differ between older people and their carers. The experience of falling has to be taken into account when designing mobile assistive technology devices as these may influence perceptions of such devices and how older people utilise them.

## 1. Introduction

The term assistive technologies (AT) covers a wide range of aids and devices designed to support older people with chronic long-term health conditions, disabilities or cognitive impairments to live at home independently. Included within the remit of AT is a plethora of devices ranging from simple mobility aids to complex computer based medical devices. Contemporary technological developments mean that mobile assistive technology (AT) devices have considerable potential—in theory at least—to contribute to the goal of enabling older people to live independently for as long as possible within their own homes by providing a range of support and alert services such as falls detection [1, 2]. Falls are a major public health problem in terms of their prevalence, morbidity, and mortality: additionally falls and fear of falling can significantly compromise the independence and quality of life of older people [3, 4]. There are a range of studies examining the detailed epidemiology of falls, potential prevention of falls, and exploring older peoples' views on falls prevention advice [5–7]. In addition, previous research into falling has aimed to develop new interventions to detect those at risk of falling, rehabilitate those who have fallen, and minimise the consequences of falls in terms of both reducing morbidity (by providing hip protectors) or reducing the time that an older person is on floor following a fall and before help arrives [5, 8–10]. In terms of falls prevention activity and interventions we can identify strategies that focus upon primary prevention (preventing falls from happening by addressing key risk factors and identifying those most “at risk”); secondary prevention (detecting falls promptly and reducing resultant injuries and other negative outcomes) and tertiary prevention (reducing the mortality/morbidity resultant from falls by prompt and effective treatment of key injuries such as hip fracture).

We can distinguish two distinct aspects of falls prevention and management where AT has a potential contribution to make: technologies that aim to prevent falls from occurring and those which focus upon the identification and notification of falls in order to reduce negative outcomes. These later devices are commonly termed “falls detectors/falls alarms” and form the focus of this paper. Such devices constitute an established assistive technology focussing upon secondary prevention whereby older people who have fallen can be identified and help summoned quickly to reduce the consequences of “long lies” on the floor [10]. The significance of these consequences should not be underestimated. It is estimated that approximately one-third of older people who fall are undetected for at least an hour [10]; there is a relationship between recovery time and the duration of the undetected lie with one study reporting that half of those who are on the floor for an hour will die within 6 months [11]. Ward et al. [12] distinguish between “generations” of falls detectors based upon the nature of the device (reactive versus proactive) and the degree of embedded intelligence within the system using the typology devised by Martin et al. [13]. First-generation falls detectors are the “traditional” falls alarm which is worn by the user and can be used to summon help in an emergency from a support centre 24 hours a day. However, these devices are entirely “reactive” and older people may not wear them as prescribed or use them in the event of an emergency [14] and many AT devices prescribed to older people or bought for them are often not appropriately used or have a low uptake [15]. In addition, these alarms usually only work within the indoor environment, and as previous research has identified there is a particular risk of older people falling outdoors due to environmental factors such as uneven pavements and weather conditions [16] as well as older peoples' fear of falling outdoors [17, 18]. For these reasons first generation falls alarm systems do not meet the needs of older people.

One response to the problems with first-generation falls monitoring devices has been the development of second-generation falls detection devices which employ embedded triaxial accelerometry to identify a fall. Whilst still reactive in nature, the use of accelerometry means that the older person does not have to activate the device. By combining data on posture, velocity, and impact the device can detect that a fall has occurred and will automatically alert the monitoring station. Such devices carry both technological challenges as well as those of acceptance by older people. Older people fall for many reasons [5], and falls may be of several types such as “heavy” falls (rapid loss of verticality), soft falls (person holds themselves up by a piece of furniture, for example, and syncopal falls (falls associated with or resulting from a loss of full consciousness) [19].

This leads to challenges and debates about the sensitivity/specificity of the different algorithms used to detect falls [20] and associated with this minimisation of the number of false negative and false positive detections. Evaluating both sensitivity and specificity is clearly important as, in order for older people to feel secure when using falls detectors, they need to be assured that they are reliable. However, due to the nature of falls, much of this proof is undertaken within laboratory situations. Illustrative of this approach is the paper by Lee and Carlisle [21] which provides proof of concept for a mobile device based upon accelerometry to detect falls events in a laboratory setting, using “young” volunteers to evaluate the sensitivity and specificity of falls detection. Based upon this evidence Lee and Carlisle then go onto to speculate that such devices are acceptable to older people in both theory and in practice. Yet, the types of device reported by Lee and Carlisle [21] have been primarily tested in laboratories with regard to reliability and performance and often with younger people rather than the intended “end users.” The capabilities of these devices for falls detection or vital signs monitoring are usually determined by the identification of the “key threats” to older peoples' independence externally that is by the analysis of epidemiological evidence or the importance of factors such as falls for health service costs rather than identifying firsthand the issues that matter most to older people. Given that use of simple alarm devices is far from universal and has limited evidence for effectiveness, there remains a knowledge deficit with regard to the acceptability of these more complex second-generation fall detection and alarm services to older people and their carers.

In this paper, we report the findings from a series of nine focus groups we conducted to (a) explore the experience and perceptions of falling amongst older people (b) explore their views of a wrist-worn AT device that could detect falls, and (c) raise broader issues about the use of mobile AT for use with older people with a particular focus on falls detection services. Our study formed part of a larger EU Framework 6 project “ENABLE—A Wearable System Supporting Service to Enable Older People to Live Well, Independently and at Ease” [22].

The ENABLE project aimed to design, develop, and test a wrist-worn device which was able to support a range of functions to support older people to live at home independently including event reminders (e.g., to take medication or attend a GP or hospital appointment), navigation and identification of a users location via GPS, control of appliances and other devices around the home, a health monitoring system, and a falls detection function which is the focus of this paper. The wrist-worn device was integrated with a mobile phone, enabling the user to get out and about, for visiting, shopping, recreation, and so forth, whilst maintaining contact for help and services [23].

ENABLE was a collaborative project between universities, voluntary/charitable groups for older people in Greece, Belgium, Czech Republic, and several AT companies. Design and development of the ENABLE device was highly user-centric with older people being involved at all stages of the system lifecycle. Central to the project was the requirement to identify the concerns and needs of older people and their carers during the concept proofing and development phases. This was achieved in two ways. A survey was undertaken across 4 EU countries (Belgium, Czech Republic, Greece, and the UK) to determine the general views of older people and their carers towards a wearable AT device and to determine the functions they would like to see provided in the device [23]. From the survey falls were identified by participants as a major concern and falls detection seen as an important element of any such device. The focus groups described in this paper were then undertaken to explore in more detail issues around falls and if (and how) an AT device could help, as well as to elicit participants' views on the device being worn on the wrist (the most favoured option in the quantitative survey).

## 2. Methods

### 2.1. Recruitment

To meet our objectives and generate focus groups who could evaluate the device functions and wearability from their experience, we recruited participants who were vulnerable to falling or the fear of falling. We invited potential participants from a number of settings, such as charity run self-help groups (Parkinson's Disease Society, Local Association for the Blind, Stroke Association), sheltered housing associations where vulnerability to falls would be expected among such groups. In addition, participants from a university research cohort of older adults were recruited in order to gain the views of those who may not currently be vulnerable to falls/fear of falling but may be so in the future. Attendees at these groups/members of the University cohort were provided with an information leaflet and reply slip to respond to the research team directly should they be interested in taking part in the study. We then provided further details and interested participants were invited to the focus groups. At the focus group meetings participants gave written consent to participate and to the recording of the group interview.

### 2.2. Data Collection

Data were collected between June and August 2008. The focus groups took place in community settings convenient to participants and were formed on the basis of recruitment site; that is, participants recruited from a stroke self-help group formed a focus group; people recruited from a vision impairment group formed another. Keeping our groups homogenous in the shared disability/chronic condition encouraged discussion of benefits and difficulties such a device may have in relation to specific impairments such as stroke or visual impairment, as well as being able to identify general issues relating to using a wearable AT falls detection device.

A prepared interview guide was used to ask participants about the difficulties they faced on a daily basis. They were then introduced to a “mock up” of the mobile AT device which would incorporate falls detection and alert services. Participants were asked about their views on the overall appearance and aesthetics of the device (including weight, size, comfort of wearing it); usability/interface (including size of buttons/screen, font size, and style of text); and overall potential challenges and opportunities such a device might bring. This paper focuses upon the potential benefits or challenges the use of this type of falls detector could bring to the lives of older people rather than on the aesthetics of the device.

Focus group interviews lasted between 35 and 50 minutes. Informed consent was taken from all participants prior to focus groups interviews and all focus groups were audiorecorded.

### 2.3. Data Analysis

Focus group interviews were transcribed verbatim from audio recordings. The software package Atlas.ti was used to store and organise data as well as facilitate the analytical process. Transcripts were analysed using a thematic analysis approach whereby data were coded into short phrases/codes, encapsulating what a particular section of data conveyed [24]. These codes were then collated to explore themes of importance to participants. Emergent themes and interpretations were discussed between the authors and any differences in interpretation resolved by discussion.

## 3. Findings

### 3.1. Participants

We conducted nine focus groups, with a total of 47 individual participants, (27 women and 20 men with an age range of 58–91 years) in the South East of England, UK (see Table 1). These groups included healthy older volunteers, older people with a range of chronic conditions and disabilities and their carers to ensure that the views of a broad range of potential users were captured.

### 3.2. Experience of Falling: Focus Group Themes

**Figure 1:**
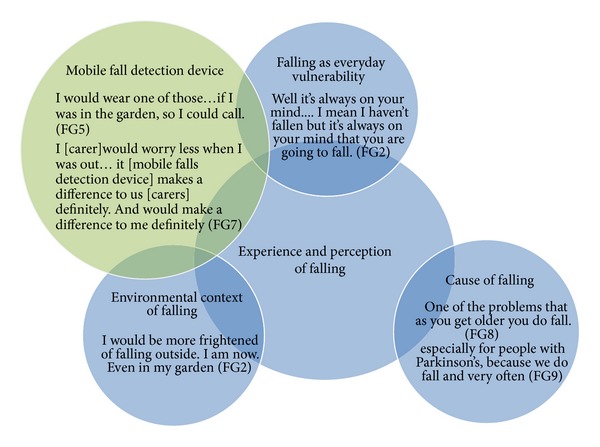
Experience of falling amongst older people and potential impact of mobile falls detection device.

Four key themes were identified relating to participants' experiences and perceptions of falling and the potential impact of a mobile falls detector: (1) cause of falling; (2) falling as an everyday vulnerability; (3) environmental context of falling; and (4) mobile fall detection device: reassurance and independence. These themes are presented in a model to illustrate how a mobile falls detection service may impact on older peoples' experience of falling across a number of different domains (see Figure 1).

### 3.3. Cause of Falling

If we are to successfully intervene, either by the development of AT devices or other programmes, to reduce falls amongst older people, it is vital that researchers understand how older people experience falls. A key narrative from our focus groups was centred on participants' understanding of the “causes” of falling. There is a consensus in the academic literature that falls are multifactorial in nature and relate to a range of intrinsic and extrinsic risk factors. In this study, participants' views on the potential cause of either their own risk, or someone close to them, falling focussed upon the causes of falling being related to pathology.

This was classified as being related to either the onset of ageing or as part of a specific disease process:

*Especially for people with Parkinson's, because we do fall and very often* (FG9).**

*One of the problems that as you get older you do fall* (FG8).**

Thus, participants in focus groups with no specific chronic illness or disability characterised ageing and/or growing older as the cause of falling. Falls were seen as a normal consequence of growing old. This contrasts to participants in a predominately “chronic illness/disability” focus group, who related their experience of falling to their specific health condition but not to the more generic process of growing older.

### 3.4. Falling as Everyday Vulnerability

Participants perceived falling as an “everyday” vulnerability alongside other factors such as living alone or increasing frailty. Falls were seen as especially pernicious in terms of increasing vulnerability reflecting their consequences which were apparent to both “fallers” and those who knew of someone who had experienced a fall. The vulnerability conferred by the real or perceived risk of falling was heightened by the lone living circumstances of many older people, with those who reported living on their own, expressing a fear of falling and this being undetected for long periods of time resulting in “long lies” on the floor. A clear advantage of the AT falls detector was that it could summon help quickly without the need for the older person to activate the system thereby reducing the risk of “long lies” in contrast to the first generation system which required the faller to activate the system:

*I think that's brilliant, no I think that's a good idea, especially for people with Parkinsons because we do fall and very often when we fall we freeze. So we are not going to press any buttons on the phone or anything else *(FG9).**

*My observation of that system is that when my sister did fall she was so shocked by the fall. She was outside on a frosty night that she forgot to use it *(FG5/6).**

### 3.5. Environmental Context of Falling

A key limitation of first-generation falls detectors is that they are limited to the user's home. Similarly the developments of domestic adaptation such as “smart” carpets that can detect falls do not extend beyond the domestic environment. The environmental context of falling was of particular interest as it linked to the previous theme of vulnerability, whilst also being identified as one “threat” that the proposed mobile falls detector could potentially remediate. Whilst the feared consequences of falls inside and outside the home were similar, the incapacity to get back up unassisted and inability to alert a carer or help centre meant that falls outside the home were seen as potentially more problematic as they were beyond the scope of conventional falls detection devices:

*I would wear one of those things, just in case. If I was in the garden, so I could call *(FG5/6).**

*Something which would tell, feed your location back to wherever, because if you've gone out for a walk and tripped and fallen down it would be useful to presumably the carer would like to know that you have fallen and go and help you. To know where you are to be helped *(FG5/6).**

### 3.6. Mobile Fall Detection Device: Reassurance and Independence

With the current state of technology both first- and second-generation falls detectors require the older person to wear them and, in the case of first-generation systems, activate them in the event of a fall. Those participants who had a “traditional” falls alarm reported that they did not always wear it but that they would wear a mobile device since it would work outside as well as within their home. Indeed, when talking about the potential mobile falls detector, participants felt the main benefit of such a device would be its ability to detect falls both inside and outside the home and alert the appropriate person without the faller needing to activate the system which was a disadvantage of first-generation devices:

*Most people who fall over, it can happen you can knock yourself out. It can happen and there is no way round that. Most of the time, nine times out of ten they can press the button* (FG7).**

*I am sure my wife would appreciate it (the AT falls detection device) … Then she would know if I have fallen down like in the area where we are living, you know, she will know that I have fallen down in the garden of the house where I live* (FG3).**

*… two doors away the women there she's fallen getting out of bed and fell against the radiator. It wasn't until a couple of days later that we discovered that she'd fallen. She couldn't activate anything* (FG8).**

The ability of the Enable device to trigger an automatic alarm when a fall was detected, was perceived as a great benefit by both the older person and carers, and as offering a clear advantage over the tradition falls alarm system, which has to be activated by the older person.

## 4. Discussion

This exploratory study provides insights into how older people, who are engaged with new technologies, perceive a wrist-worn AT device for falls detection and its potential benefits and disadvantages. Whilst there are studies looking at AT more generally [25] or at first-generation falls detectors previous studies of second-generation falls detectors have predominantly been undertaken with younger people or, if they have included older people, have not been engaged with the perspectives of their carer(s) [12].

For carers the major advantage of the device was focussed around the notion of reassurance, and this finding is supported by previous research into the perceived benefits of telehealth and assistive technology solutions [26, 27]. Older people living with a chronic illness or disability identified improvements in wellbeing and potential enhanced safety afforded by the emergency and medical functions as the key advantages of the device in early evaluations of telehealth and assistive technological devices [28, 29].

Previous research into a number of assistive technology devices including hearing aids [29], emergency alarm pendants [30], and falls detectors [31] found that participants were reluctant to wear the device during waking hours because of the physical attributes of the devices and perception of social stigmatisation. As one of participants in FG5 stated “*but you see if they have a fall as you say with a pendant, they can always take them off*.” There are other reasons for lack of use of fall detectors including concern for invasion of privacy [32–34]. It has also been reported that some users avoid wearing their fall detector as it is uncomfortable or produces false alarms [15, 35]. Brownsell and Hawley [31] suggested that some older people may not be inclined to adopt a product that would alert their informal and formal carers to falls, fearing institutionalisation. The lack of control over whether an alert is sent has also been postulated to be a factor affecting fall detector use as users do not want to “bother” anyone [15].

Whilst the participants in our study did not voice any concerns regarding being stigmatised when wearing an AT device, they did express issues around vulnerability, when wearing the device in public, which is diametrically opposed to the devices aim of increasing perceived reassurance and safety. Parker et al. [22] also found that older people were concerned about the requirement to wear a sensor at all times and the constant monitoring of their movement, due to anxieties regarding invasion of personal privacy. Our data indicate that older people perceived that the wrist-worn falls detector could address two key deficiencies in traditional falls alarms: overcoming the limitation of devices that only work indoors and the vulnerability resultant from falling and being unable to summon help (which the device can do outside and automatically). However, if the older person does not perceive that they are at risk of falling and, therefore, in need of a fall detector, then, no matter how good the technology is, it will not be adopted. Including potential end users in the development of an AT device and obtaining feedback on the device throughout the development cycle can contribute significantly to ensuring a user-friendly design and the fact that end users' needs and concerns are taken into consideration, thus potentially improving uptake rates and adherence to such devices [36].

## 5. Conclusion

We fully acknowledge the limitations of our data which were collected as part of a larger project exploring the perceptions and views of older people and their carers on a specific device rather than exploring their attitudes to assistive technology and telehealth in general. Participants were volunteers who were existing mobile phone users and as such may not reflect the use of current technologies amongst the general older population, particularly the oldest old or those with severe disabilities. However, using the example of a falls detection system, we have demonstrated that although laboratory-based evaluations of such devices can be technically successful, the acceptability of such devices to older people cannot simply be extrapolated from such trials. Such technology raises concerns amongst older people, which need to be considered in order to ensure that the objectives of AT devices can be achieved. Furthermore, we have demonstrated that carers and older people articulate different perspectives upon such systems. Carers are seeking reassurance from such devices, but this may be achieved at the cost of increasing the perceived vulnerability and loss of privacy of older people. Further work is required in order to ensure that the voices of older people and their carers are central to the development of technologies to enable older people to live at home independently.

## Figures and Tables

**Table 1:** Participant characteristics.

	Age range	Gender	Disability/Chronic illness	Living setup
FG1	55–80 years	1 woman, 3 men	Stroke	All with partner/spouse
FG2	68–89 years	4 women, 2 men	Vision impaired	Unknown
FG3	73–88 years	5 women, 2 men	Mild dementia, vascular disease, Parkinson's disease	On own: 3, with partner/spouse/relative: 4
FG4	64–98 years	2 women, 2 men	Arthritis, Parkinson's disease	On own: 4
FG5	62–85 years	2 women, 3 men	None	On own: 2, with partner/spouse: 3
FG6	65–76 years	4 women, 3 men	Arthritis, hearing impaired	All live with partner/spouse
FG7	54–77 years	4 women, 3 men	Parkinson's Disease, stroke	All live with partner/spouse
FG8	72–84 years	3 women, 1 man	Arthritis	All on own
FG9	63–66 years	2 women, 1 man	Parkinson's disease	On own: 1, with partner/spouse: 2
